# Browsing or buying: A serial mediation analysis of consumer’s online purchase intentions in times of COVID-19 pandemic

**DOI:** 10.3389/fpsyg.2022.1008983

**Published:** 2022-10-21

**Authors:** Hina Yaqub Bhatti, Madiha Bint E. Riaz, Shazia Nauman, Muhammad Ashfaq

**Affiliations:** ^1^Riphah School of Business and Management, Riphah International University, Lahore, Pakistan; ^2^Research Institute of Business Analytics and Supply Chain Management, College of Management, Shenzhen University, Shenzhen, China

**Keywords:** perceived usefulness, personal innovativeness, internet browsing, online purchase intentions, attitude towards online purchasing

## Abstract

The role of digitization and globalization have changed consumers’ online buying behaviors, specifically in the times of the COVID-19 pandemic crisis. This seriously influences the online retail industry in developing countries that are already struggling to move toward digital trading through e-business. Pakistan being a developing country is no exception, and it is, therefore, pertinent to examine factors that contribute to digital trading. Employing theories of reasoned action and the technology acceptance model, this study aims to investigate how personal innovativeness and perceived usefulness impact consumers’ online purchase intentions through a serial mediational model. The data were collected through an online survey from 410 respondents. Structural Equation Modeling (SEM) was used to test the proposed model. This study showed significant results for the direct effect of personal innovativeness and perceived usefulness on online purchase intentions as well as the indirect serial effect *via* internet browsing and attitude toward online purchasing. The study results have some important practical implications for selling firms, especially in the times of COVID-19. The study suggests that online retailers should be more responsive to the aforementioned factors to facilitate consumers to spend more time browsing, which influences consumers’ interest and intention to make online purchases. As the social distancing and lockdown approaches were implemented in Pakistan and other parts of the world, the trend toward online purchases has increased. Due to this shift in the overall purchasing behavior of consumers and the potential for strong growth in e-commerce, organizations need to consider the post-COVID situation to expand their business in an online platform for addressing the future pandemic crisis.

## Introduction

The online retail industry has performed as an emerging market to provide a platform for local and international transactions (Lim et al., 2016). Over the last decade, online transactions have been rapidly increasing *via* the development of internet technology. According to Statista (2021), the total revenue of the E-commerce market is 4.9 trillion US dollars and is expected to grow 50 percent in the next 4 years, amounting to 7.4 trillion dollars by 2025. Concerning Pakistan, the total revenue of the E-commerce market is US$6 billion in 2021 making it the 37th largest E-commerce market and it is expected to grow by 7% in the next 4 years (eCommerce, 2021). The role of the online retail industry has been boosted, especially during the COVID-19 pandemic, where retailers have exerted much effort in establishing, improving, and promoting their respective online store commerce (Koch et al., 2020). The Internet penetration rate is higher in developed countries in comparison with developing countries (Yavich et al., 2019). However, the market trend demonstrates that online shopping has tremendous growth during the COVID-19 pandemic era and shapes consumers’ online purchasing behavior.

The online trends developed during COVID-19 might go on for a long time imposing serious concerns for brick-and-mortar stores due to the rapid growth of online trade. Despite the extensive research on online purchase behaviors, the factors that impact online purchase intentions, especially considering the times of the COVID-19 pandemic still need to be investigated (Al-Hassani et al., 2020; Hasanat et al., 2020; Akar, 2021). Numerous factors impact consumers’ online purchase intentions, and the increased level of uncertainty during the COVID-19 pandemic thereby offers a peculiar opportunity to investigate online purchase behaviors (Ait Youssef et al., 2020; Hasanat et al., 2020; Rahman et al., 2020). The role of online purchase or shopping in routine life provides a better comparison of products easily, quickly, and effectively by using some electronic clicks (Jiang et al., 2013; Nazir and Haq, 2018). Based on the statistical evidence, there are 4.66 billion internet users worldwide, including 59% of the worldwide population, Moreover, considering Pakistan, there are 61.34 million internet users (Statista, 2021), and the population of Pakistan is 225.199 million (Worldbank, 2021).

Additionally, the COVID-19 pandemic has brought a significant change in consumer behavior worldwide, and Pakistan is no exception to it. The prospects of growth *via* online sales seem to be huge and promising (Alhaimer, 2022). The Pakistan e-commerce market is displaying an average growth rate of 6.09% *per annum* (2022–2025); furthermore, it has been reported that the E-commerce market of Pakistan will reach about 9,153 millions US dollars by 2025 (Statista, 2022). In addition to that, the growth of the online market in the Asia Pacific region like Pakistan is attributed to various benefits such as changing consumers’ lifestyles, causing an increased penetration in communication and information technology (Narang and Trivedi, 2016). Such online behaviors have been the epicenter of research mainly in developed countries (Gao and Li, 2019) and have received little attention in developing countries (Prashar et al., 2015; Aldousari et al., 2016), like Pakistan (Abbasi et al., 2019). Extant literature suggests a need to study the crucial factors that can encourage consumers in developing countries to embrace the e-commerce market (Peña-García et al., 2020) and our study fills this gap.

Though the research on online purchase intentions is extensive, however, understanding of the factors influencing online purchase intentions, specifically during the COVID-19 pandemic, remains scant (Al-Hassani et al., 2020; Hasanat et al., 2020; Rahman et al., 2020). Dharmesti et al. (2019) called for further research on online purchase behaviors in their study on understanding online purchase intentions among millennials’. Another study highlighted the opportunity to investigate further factors that can potentially impact the consumer’s attitude toward online shopping (Chetioui et al., 2020). It can be stated that the role of browsing is well investigated in the traditional retail setting stores (Xia, 2010; Helmefalk, 2019) however, its role in an online setting needs to be further explored (Zhang et al., 2018).

Earlier research on the buying behaviors of consumers has focused mainly on either internet browsing or attitude toward online purchasing. For instance, Lu et al. (2020), unveiled that internet browsing increases online purchase intentions. Furthermore, Zhang et al. (2018) have focused primarily on how utilitarian (e.g., Perceived Usefulness) and hedonic values (e.g., Personal Innovativeness) lead to internet browsing, and that ultimately leads to buying behavior. In another research, Park et al. (2012) explained the mediating role of browsing between different product attributes and buying behavior.

Alhaimer (2022), studied how attitude act as a mediator in the relationship between various risk factors and online purchase behaviors. Additionally, Huseynov and Özkan Yıldırım (2019), investigated the relationship of attitude to online shopping with online purchase intentions considering online consumer segmentation. In another research, Hwang et al. (2021), explained the relationship of attitude with functional, social, and hedonic motivations and online purchase intentions. Therefore, the current study is novel in its contribution to the existing literature as it attempts to conduct serial mediation to test a causal chain linking the mediators with a specified direction of causal flow and then their impact on dependent variable (Hayes, 2012, p. 14). For example, perceived usefulness and personal innovativeness increase internet browsing which could increase attitude toward online purchasing and thus increase the online purchase intentions (i.e., perceived usefulness and personal innovativeness → internet browsing → attitude toward online purchasing → online purchase intentions).

Accordingly, our study investigates four research questions (RQ): RQ1: How do perceived usefulness and personal innovativeness impact online purchase intentions? RQ2: Is there any mediation of internet browsing between the relationship of perceived usefulness, personal innovativeness, and online purchase intentions? RQ3: Is there any mediation of attitude toward online shopping between the relationship of perceived usefulness, personal innovativeness, and online purchase intentions? RQ4: Is there any serial mediation of internet browsing and attitude toward an online purchase between the relationship of perceived usefulness, personal innovativeness, and online purchase intentions?

To provide a theoretical lens for examining antecedents of online purchase intentions during the COVID-19 pandemic, the current study draws on the work of two basic theoretical models, i.e., the theory of reasoned action (TRA) and the technology acceptance model (TAM). TRA explains the psychological process that lays the foundational basis for a consumer’s decision-making (Fishbein et al., 1980). TAM describes that the behavioral intention to use a certain new technology is determined by the person’s attitude toward that technology. Referring to this, TAM originally identified two basic determinants of attitude toward technology in the form of perceived usefulness and ease of use. Though TAM is considered to be a viable model for examining the consumer’s acceptance of new systems and technologies (y Monsuwé et al., 2004; Gounaris and Koritos, 2008), it is still pertinent to extend this model by incorporating additional factors to define more specific drivers of consumer’s acceptance toward internet technology (Ait Youssef et al., 2020). Hence, the current study attempts to extend the model firstly by analyzing how Perceived Usefulness (PU) and Personal Innovativeness (PI) affect Online Purchase Intentions (OPI). Secondly, the novelty of the current research lies in its contribution to the underlying mechanism through which perceived usefulness and personal innovativeness impact online purchase intentions. Third, this study attempts to extend the existing literature by introducing serial mediation of internet browsing and attitude toward online purchasing between the relationship of perceived usefulness, personal innovativeness, and online purchase intentions. Lastly, it answers the calls for research in a developing country context (Ur Rahman et al., 2018; Abbasi et al., 2019).

## Literature review

### Perceived usefulness and online purchase intentions

Purchase intentions are considered to be the key predictor of actual purchase behavior (Montaño and Kasprzyk, 2015). Online purchase intentions can be identified as the extent of willingness to which consumers desire to buy the product online (Ajzen, 1991; Pavlou, 2003). Lack of intent to purchase online acts as a key barrier to the development of E-commerce (Peña-García et al., 2020). It is, thereby, pertinent to further investigate online purchase intentions. Earlier research on the determinants of purchase intentions based on the framework of e-commerce highlights the importance of utilitarian motives along with hedonic motives (Van der Heijden, 2004). Utilitarian motivation relates to the usefulness of behavior, whereas hedonic motivation relates to the pleasurable experience gained from the behavior (Holbrook and Hirschman, 1982; Batra and Ahtola, 1991). Based on such motivations, this study investigates how perceived usefulness and personal innovativeness influence online purchase intentions.

Consumer characteristics substantially affect their behavior (Mount et al., 2005). Behavioral intentions toward the online environment are widely supported by the TAM (Davis, 1989), and technology is said to be successful if it creates the value of usefulness for a customer. Perceived usefulness refers to the consumer’s perception regarding the enhancement of his/her performance through the use of technology (Davis, 1989, 1993). It has a significant role in consumers’ online purchase decisions (Singh et al., 2018). Applying this definition to the context of online purchases, *usefulness* refers to the consumer’s belief that Internet use will enhance consumers’ performance or productivity, thereby improving their shopping experience (y Monsuwé et al., 2004). Precisely, the perceived benefits of shopping online can be summarized as perceived usefulness (Moslehpour et al., 2018). The effortlessness in shopping online rather than shopping from a traditional store also contributes to consumers’ perceived usefulness. Additionally, having a discounted price in addition to searching for low costs can also be useful for consumers. This perceived usefulness of consumers can impact their willingness to purchase online (Moslehpour et al., 2018).

Numerous studies explained the relationship between perceived usefulness, attitude, and intention (Davis, 1989; Lee et al., 2003; Chiu et al., 2005; Bae and Lee, 2011; Zhang et al., 2018; Rehman et al., 2019). In one study, the findings of Dash and Saji (2006) showed a significant positive association between perceived usefulness and purchase intention of B2C online buyers in an Indian context. In another study, Koufaris (2002) showed a positive relationship between perceived usefulness and purchase intentions by analyzing unplanned purchases in an e-commerce context. Based on the TAM, the current study attempts to explain that the perceived usefulness of consumer specifically in the times of COVID-19 aid in the acceptance of new technology by increasing online purchase intentions. Hence, the current study proposes that,

*H1 (a):* Perceived usefulness is positively related to online purchase intentions.

### Personal innovativeness and online purchase intentions

The novelty in an online shopping atmosphere prompts innovative customers first for the actual purchase (Boyle and Ruppel, 2006). The belief of considering the Internet as a new channel for buying products and considering that it can offer advantages such as ease of use, lower prices, and more selection options will tend to attract innovative customers. An individual’s innovative behavior is considered a key element in accepting new technologies (Brancheau and Wetherbe, 1990; Wu et al., 2014).

Innovativeness as an individual’s personality trait is considered to be one of the determinants in the adoption of the internet as a shopping medium that can lead to purchases (Citrin et al., 2000). Hence, personal innovativeness can be explained as the behavior of the consumer that reflects a willingness to adopt new things, specifically technology development, and build strong attention to the absorption of that application with a cognitive attitude (Cheong and Park, 2005). Various studies in e-commerce context have revealed the importance of personal innovativeness on online purchase intentions (Park and Jun, 2003; San Martín and Herrero, 2012; Ha and Im, 2014; Srinivasan, 2015; Thakur and Srivastava, 2015; Nagar and Gandotra, 2016; Dewi et al., 2020). Specifically, the times of COVID-19 have compelled consumers to adopt online mediums for their purchases due to the motivation in form of personal innovativeness (Hwang et al., 2021).

Based on the notion of the theory of reasoned action, it is suggested that highly innovative individuals are more prone to the acceptance of new technologies (Lee et al., 2007). Therefore, it is proposed that,

*H1 (b):* Personal innovativeness is positively related to online purchase intentions.

### Mediating role of internet browsing

Growth in internet penetration has a great impact on consumers’ preferences for using it as a tool for browsing and searching for product information (Moe and Fader, 2004; Soopramanien and Robertson, 2007). Earlier studies showed that people browse online not only for information gathering but also for fun (Floh and Madlberger, 2013). People’s buying behavior significantly depends upon utilitarian and hedonic motives (Pöyry et al., 2013; Zhang et al., 2018). Utilitarian motives (like perceived usefulness) lead consumers to browse for product information and to optimize the outcomes of future purchases (Wang, 2010). Consumers thus put a great emphasis on browsing for information collection while shopping online (Smith and Sivakumar, 2004). Furthermore, the hedonic motives make consumers gratified from the browsing process where they not only browse for information but also seek enjoyment (Korgaonkar and Bellenger, 1980). Online browsing hence contributes to people’s explorative information search behavior (Moe, 2003). The basic aim of browsing is to buy the products efficiently, thereby saving the price and consumers’ time (Overby and Lee, 2006).

Consumers’ perceived usefulness can influence online browsing behaviors in terms of convenience, sales, or ease of purchase. Consumers who seek convenience for online transactions browse various websites (Bhatnagar et al., 2000). In another study, it was revealed that value motivation acts as a significant contributor to online browsing (Ono et al., 2012). This value motivation relates to the perceived benefits of sales, discounts, and bargaining (Arnold and Reynolds, 2003). Consumers’ online browsing is perceived to be useful for customers as it allows simultaneous search and purchase options with just a few clicks in comparison to the offline context, which requires a series of steps (Demangeot and Broderick, 2009; Kumar et al., 2010; Siren, 2013).

Furthermore, a consumer’s innovative style also contributes to his/her online browsing behavior. Innovative consumers browse information relating to new product launches (Manning et al., 1995). Another study conducted by Wang et al. (2006) revealed how innovative and cognitive styles impact consumers’ website behavior. An adventurous stimulus in consumers for seeking new things through the use of technology prompts their browsing behavior (Ono et al., 2012). Thus, the consumer’s perceived innovativeness as a personality trait exhibits a substantial role in motivating consumers to use new technologies through browsing (Khare et al., 2010).

Another study revealed a significant relationship between information search and online purchase intentions (Krithika and Rajini, 2017). Additionally, a study based on the online shopping behaviors of young consumers reported that their information search behavior significantly influences their purchase intentions (Dharmesti et al., 2019). This search behavior in terms of browsing affects consumers’ online purchase intentions where consumers search about goods and services, select the product and supplier and order the goods and services (Hajli et al., 2017). It is reported that purchase decisions depend upon the browsing characteristics, i.e., the number of pages viewed and the time spent on browsing (Mallapragada et al., 2016). Earlier research on online shopping signaled that online purchase behavior could be anticipated through the incorporation of browsing characteristics (Bucklin and Sismeiro, 2003; Montgomery et al., 2004). Furthermore, as consumers spent more time on internet browsing during the times of COVID-19, therefore, the current study seeks to contribute to the TAM by investigating how perceived usefulness and personal innovativeness impact internet browsing, which ultimately leads to online purchase intentions and thus explains the mechanism for the acceptance of new technology. Therefore, we posit the following hypotheses:

*H2 (a):* Perceived usefulness is positively related to online purchase intentions via internet browsing.

*H2 (b):* Personal innovativeness is positively related to online purchase intentions via internet browsing.

### Mediating role of attitude toward online shopping

Contrary to the traditional retail store environment, the online purchase environment integrates the sales process on a single channel (Wang et al., 2009). Unlike traditional physical stores, consumers cannot have the tangible experience while purchasing online, but they get the benefit in the form of increased convenience where they are free from geographic limits as well as time limits. Consumer perceptions and their experiences toward such benefits and risks shape their attitude toward online shopping (Wu, 2003; Ahn et al., 2007). Consumer attitude can greatly influence their purchase decisions (Wu, 2003). However, earlier studies on attitudes toward online purchases are limited to technology and consumer demographics (Lissitsa and Kol, 2016). Furthermore, COVID-19 have shaped consumers’ attitude toward online purchasing (Hwang et al., 2021) and therefore it is pertinent to investigate it further.

Attitude is recognized as a person’s inclination of acting in a certain way toward a concept or object (Doob, 1947). Attitude toward online shopping is a multidimensional construct based on the consumer’s acceptance of the internet as a shopping medium and consumers’ attitude toward a specific online store (Li and Zhang, 2002; Soopramanien and Robertson, 2007). Based on the view of the TRA, attitude is considered to be an immediate cause of intention to perform any behavior (Fishbein et al., 1980). A positive attitude of consumers increases their purchase intentions (Seock and Norton, 2007). Motivations behind consumers’ online shopping attitude span both utilitarian and hedonic extents. Some online buyers are considered problem solvers; others can be described as seekers of “fun, fantasy, arousal, sensory stimulation or enjoyment” (y Monsuwé et al., 2004).

Based on the utilitarian perspective, consumers adopt online shopping if they perceive superior benefits over the traditional shopping medium (Choudhury and Karahanna, 2008). This superior benefit can be in the form of usefulness achieved through lower-cost minimal time and effort (Choudhury and Karahanna, 2008; Hsiao, 2009). Arora and Aggarwal (2018) also suggested three main benefits linked to online shopping, namely “price, convenience, and recreational benefits.” This perceived usefulness impacts consumers’ attitudes toward online shopping. Referring to the TAM model, “usefulness” refers to the consumer’s perceptions that internet use as a shopping medium will enhance the outcome of the shopping experience (Davis, 1989, 1993). Hence, these perceptions impact consumer attitudes toward online shopping, which ultimately influences consumers’ purchase intentions toward online shopping (Pandey and Parmar, 2019).

Various researchers investigated the role of personal innovativeness in new technology acceptance (Leonard-Barton and Deschamps, 1988; Agarwal and Prasad, 1998; Bellman et al., 1999; Im et al., 2003; O’cass and Fenech, 2003). An individual’s innovativeness influences how consumers use online shopping. Jones et al. (2002) described that the extent of a consumer’s technology use behavior is influenced by the level of his/her innovativeness. In another study, Leonard-Barton and Deschamps (1988) stated that highly innovative users with high managerial support are more likely to use a certain technology while users at the low innovative level with no managerial support are less likely to accept the use of a certain technology. Hence, the user’s perceptions and intentions toward internet use are highly influenced by innovative dispositions (Agarwal and Prasad, 1998). It is suggested that innovative individuals tend to establish a more positive attitude toward new technologies (O’cass and Fenech, 2003), i.e., an attitude toward online shopping, which resultantly enhances their online purchase intentions. Therefore, we posit the following hypotheses:

*H3 (a):* Perceived usefulness is positively related to online purchase intentions via attitude toward online shopping.

*H3 (b):* Personal innovativeness is positively related to online purchase intentions via attitude toward online shopping.

### Serial mediation of internet browsing and attitude toward online shopping

Madden et al. (1992) theory of reasoned action (TRA) is adopted as a widely used attitude model for the explanation of consumer buying behavior (Lee et al., 2007). The theory of reasoned action proposes that an attitude is an immediate cause of performing a specific behavior (Fishbein et al., 1980). TRA describes the psychological process that leads to a consumer’s decision (Fishbein et al., 1980). It has been significantly used to identify the behaviors to behave in a certain way as determined by a person’s attitude (Yeo et al., 2017). Hence, technology use influences consumers’ attitudes in various ways (Alcántara-Pilar et al., 2018).

It has become quite easy for consumers to find products and relevant information through web browsing, which reduces their costs and increases convenience for consumers (Kacen et al., 2013). The interactive nature of the Internet and the Web offers many opportunities to increase the efficiency of online shopping behavior by improving the availability of product information, enabling multi-attribute comparisons, and reducing buyer’s search costs (Alba et al., 1997). Within the Technology acceptance model, interactive media is thought to shed light on the more instrumental aspects of online shopping. Some consumers may shop primarily for utilitarian purposes, while others for adventurous or enjoyable purposes. Both these purposes can ultimately affect consumers’ attitudes toward online shopping (Childers et al., 2001).

**Figure 1:**
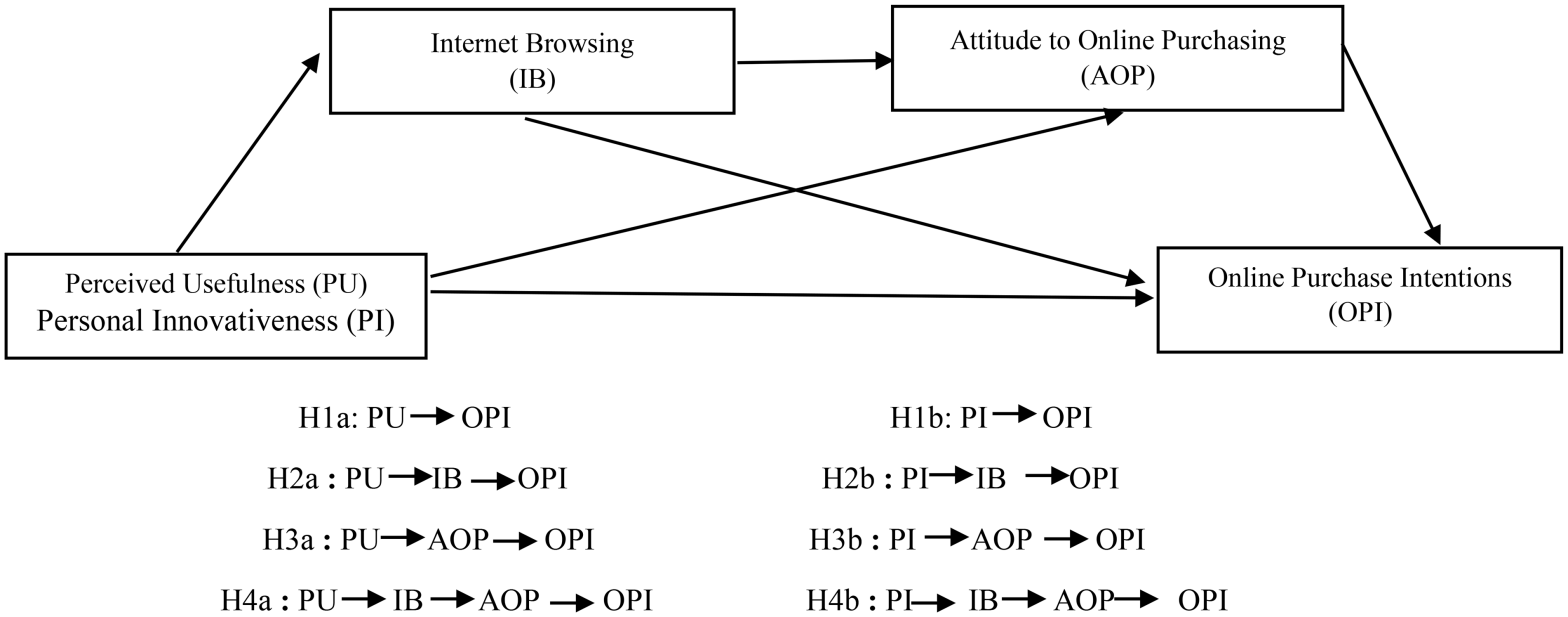
Proposed model. Authors constructed.

In a study by Teo (2002), various factors based on the consumer’s search have been identified to create a consumer’s attitude toward online shopping. He described that consumers’ active browsing of the internet has become part of their lifestyle which supports our study’s argument that internet browsing aid in shaping consumers’ attitude toward online purchase intentions and that ultimately stimulates the online purchase intentions (Figure 1).

When consumers shop online, they pass through the stages of browsing, where various factors influence the aspects of the purchase process (Mallapragada et al., 2016). Hence, consumers engaged in the online browsing of websites go through several website features where they may visit FAQ sections or the community sections to get additional product details (Bart et al., 2005). These features may assist the consumer in better navigation through various website maps, designs, or other presentation elements (Hong et al., 2004). Thus, the browsing process eventually influences consumers’ shopping outcomes. Hence, the current study postulates that online browsing (either caused by the motivation of perceived usefulness or personal innovativeness) shapes the consumer’s attitude toward online shopping, which ultimately impacts their online purchase intentions (Küster et al., 2016). Therefore, we posit the following hypotheses:

*H4 (a):* Internet browsing and attitude toward online purchase serially mediate the relationship between perceived usefulness and online purchase intentions.

*H4 (b):* Internet browsing and attitude toward online purchase serially mediate the relationship between personal innovativeness and online purchase intentions.

## Methodology

### Sampling and data collection

This survey was circulated to Pakistani consumers who have been purchasing online products during COVID-19 from different cities in Pakistan. Convenience sampling was used for this research. The minimum sample size was calculated by the thumb rule (VanVoorhis and Morgan, 2007). The thumb rule stated that the sample size for this research data set should be (5 variables × 30 = 150). The purpose of this rule is to ensure enough data is collected to achieve an acceptable level of statistical power. The online questionnaire form was available on google form for almost 2–3 months. The first part of the questionnaire included demographic factors such as gender, age, educational background, and family monthly income (PKR). The second part was comprised of questions like “most frequent items that consumers have purchased online during COVID-19,” “how long you have been purchasing online products,” and “which electronic devices are you using during online purchases.”

Of all 410 consumer respondents, 273 were male (66.6%), and 137 were female (33.4%). 67.3% were between 18 and 29, 32.2% were between 30 and 49, 0.5% were over 50 years old. 18.1% of participants were elementary college graduates, 28.0% were Bachelor’s degree holders, 48.5% had master’s degrees, and 5.4% were Ph.D. graduates. 10.0% of individuals had family income levels below Rs. 25,000, 23.9% had income levels between Rs. 25,000–50,000, 18.0% had income levels between Rs. 50,000–75,000, 23.4% had income levels between Rs. 75,000–100,000, and 24.6% had income levels above Rs. 100,000.49.5% of consumers took an interest in online shopping, 29.5% of consumers bought or got online food, and 18.0% of consumers also purchased a ticket for transport facilities. 2.9% of consumers opt for other facilities such as home and mobile accessories. In this study, we observed that 23.9% of consumers have been using online purchases for <3 months. 15.1% of consumers have been using online shopping for 3–6 months, 16.8% of consumers have been using online shopping for 7–12 months, 15.1% of consumers have been using online shopping for 13 months–2 years, and 29.0% consumers have been using this advance technology for online purchase for more than 2 years. This study also noticed that 93.2% of consumers shop through cell phones/mobiles, 5.6% of consumers use PC/desktops, and 1.2% of consumers use tablet devices for online shopping.

## Measures

Most of the studies conducted in Pakistan have indicated that English is an appropriate and understandable medium in the country; hence there is no need to translate questionnaires into national languages (Urdu; Fatima et al., 2020).

All variables were measured on scale anchors ranging from 1 = “Strongly disagree” to 5 = “Strongly Agree.”

### Personal innovativeness

Personal innovativeness was measured with a 6-item scale developed by McKnight et al. (2002). A sample item is “I like to explore new websites.” (Cronbach’s alpha = 0.893).

### Perceived usefulness

Perceived usefulness was measured with a 3-item scale developed by Taylor and Todd (1995). A sample item is “The Internet would be useful in my purchasing” (Cronbach’s alpha = 0.702).

### Attitude toward online purchasing

Attitude toward online purchasing was measured with a 4-item scale adopted by Taylor and Todd (1995). A sample item is “The use of online purchasing is a good idea” (Cronbach’s alpha = 0.885).

### Internet browsing

Internet browsing was measured with a 2-item scale developed by Beatty and Ferrell (1998). A sample item is “The percentage of time I Spend just looking around on the online group shopping website is fairly high” (Cronbach’s alpha = 0.744).

### Online purchase intentions

Online purchase intentions were assessed with a 4-item scale developed by Taylor and Todd (1995). A sample item is “Given the opportunity, I will use the online purchasing” (Cronbach’s alpha = 0.727).

## Assessment of common method variance bias

We used Harmon’s one-factor test to identify the common method variance bias (Podsakoff et al., 2003). The findings showed that the total variance of the first factor is 31.05%, well below the threshold value of 50%, suggesting that there is no issue of common method variance.

## Results

The descriptive characteristics and correlations are reported in Table 1. Confirmatory factor analysis is reported in Table 2. Table 3 represents serial multiple mediation analysis.

**Table 1:** Descriptive statistics, zero-order correlations, and reliability.

	Variables	Mean	SD	1	2	3	4	5
1	Perceived usefulness	3.56	0.70	(0.70)				
2	Personal innovativeness	3.42	0.87	0.41^**^	(0.89)			
3	Internet browsing	3.32	0.80	0.35^**^	0.31^**^	(0.74)		
4	Attitude toward online purchasing	3.52	0.87	0.46^**^	0.31^**^	0.34^**^	(0.89)	
5	Online purchase intentions	3.71	0.65	0.45^**^	0.34^**^	0.11^*^	0.44^**^	(0.73)

*N* = 410. *p<0.05; **p<0.01; ***p<0.001.

**Table 2:** Model fit and confirmatory factor analysis results of the measurement model.

Construct	Item	Factor loading	Cronbach’s alpha	CR	AVE
Perceived usefulness	PU1	0.77	0.79	0.78	0.65
	PU2	0.68			
	PU3	0.78			
Personal innovativeness	PI1	0.83	0.91	0.91	0.65
	PI2	0.74			
	PI3	0.78			
	PI4	0.90			
	PI5	0.81			
	PI6	0.80			
Internet browsing	IB1	0.73	0.77	0.78	0.64
	IB2	0.86			
Attitude toward online purchasing	AOP1	0.69	0.88	0.88	0.64
	AOP2	0.80			
	AOP3	0.82			
	AOP4	0.89			
Online purchase intentions	OPI1	0.71	0.84	0.84	0.57
	OPI2	0.84			
	OPI3	0.81			
	OPI4	0.65			
**Model**	***χ*** ^2^ **/df**	**CFI**		**RMESA**	
Research model	2.52	0.95		0.06	

**Table 3:** Tests of direct and indirect effects of model 6.

Effect	Standard error	Coefficient	95% confidence interval (CI)
**Direct effect**			
PU → OPI	0.04	0.32	(0.19, 0.44)
PI → OPI	0.03	0.18	(0.11, 0.26)
**Indirect effect**			
PU → IB → OPI	0.02	−0.04	(−0.08, −0.01)
PI → IB → OPI	0.14	−0.02	(−0.06, −0.01)
PU → AOP → OPI	0.03	0.11	(0.07, 0.18)
PI → AOP → OPI	0.23	0.07	(0.03, 0.12)
PU → IB → AOP → OPI	0.01	0.02	(0.01, 0.05)
PI → IB → AOP → OPI	0.01	0.03	(0.01, 0.05)
**Total effect**			
PU → OPI	0.04	0.42	(0.30, 0.53)
PI → OPI	0.03	0.25	(0.17, 0.32)

PU, Perceived Usefulness; PI, Personal Innovativeness; IB, Internet Browsing; AT, Attitude to Online Purchasing; AOP, Online Purchase Intentions OPI.

### Descriptive statistics and correlations

Table 1 describes the descriptive statistics and correlations among all variables. Descriptive statistics and correlation analysis were performed in SPSS between perceived usefulness, personal innovativeness, internet browsing, attitude toward online purchasing, and online purchase intentions. The result is displayed in Table 1.

### Summary of validity and reliability test

Table 2 shows the findings of the validity and reliability test. For confirmatory factor analysis, the SEM model has been done to justify the discriminant validity of all variables. We have run a full measurement model comprising the five variables (i.e., perceived usefulness, personal innovativeness, Internet browsing, attitude toward online purchasing, and intention toward online purchasing). The result summary of the full measurement model showed that each item of all variables had a factor loading of 0.50 above (Gerbing and Anderson, 1988).

The discriminant validity of these data was investigated by Fornell and Larcker (1981). The construct reliability was evaluated by Composite Reliability (CR) and Cronbach’s Alpha (CA). Table 2 results showed that the values of CR and CA were >0.70 and all the variables lie within the acceptable range recommended by Nunnally (1978) and Hair et al. (2017). The model fit results revealed (CMIN/DF = 2.52, CFI = 0.95, RMSEA = 0.06). According to Hu and Bentler (1998), all these values are acceptable for the model to fit.

Table 2 also represents Cronbach’s alpha values. All the variables proposed in the conceptual model have shown significant results. All scales of the reliabilities are above the cutoff point of 0.70.

### Serial multiple mediation models

**Figure 2:**
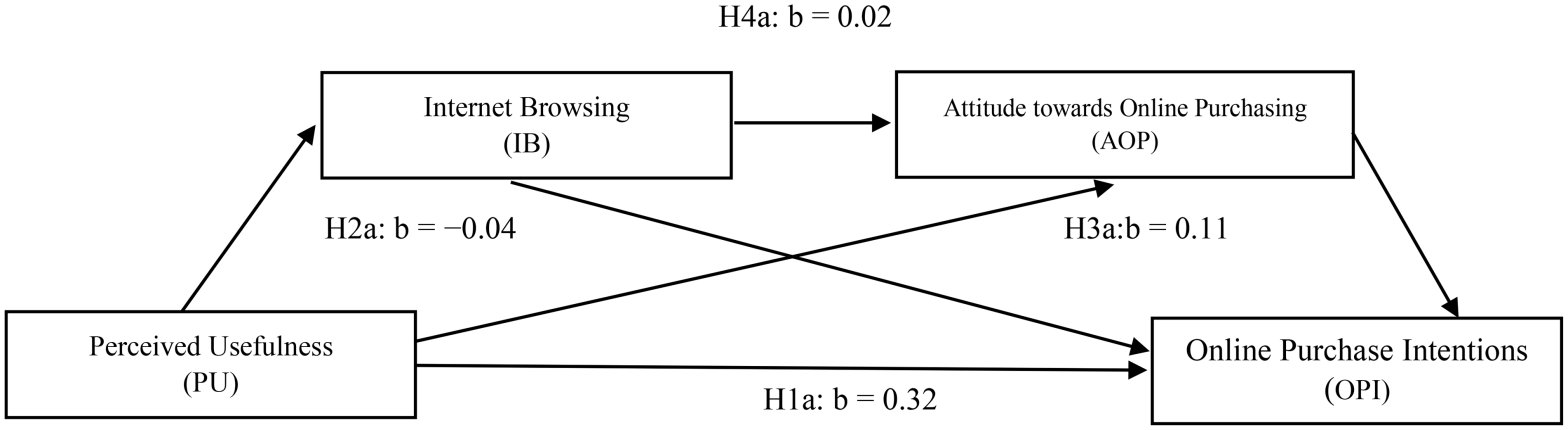
Serial multiple mediation models include Perceived Usefulness (PU), Internet browsing (IB), Attitude toward online purchasing (AOP), and Online purchase intentions (OPI). Authors constructed.

To test the hypothesis of the proposed model (Figure 2) whether internet browsing and attitude toward online purchasing serially mediated effects of perceived usefulness on online purchase intentions (serial mediation Model 6) by Hayes and Preacher (2013), we used bootstrap methods. Figure 2 illustrates all possible paths and their related coefficient values. We found that the first path (H1a) significantly affected online purchase intentions with coefficient value, *b* = 0.32, *p* < 0.001, and supported H1a.

The statistical significance of the indirect effects in the model tested in the current research was examined on 5,000 bootstrap samples. Estimates were taken within a 95% confidence interval, and bias-corrected results are presented in Table 3. It shows that internet browsing significantly mediated perceived usefulness and online purchase intentions, *b* = −0.04, SE = 0.02, 95% CI = −0.17, and − 0.02 (H2a supported). Likewise, the indirect effect through attitude toward online purchasing between perceived usefulness and online purchase intentions was significant, *b* = 0.11, SE = 0.03, 95% CI = 0.07, and 0.08, which supported H3a. For testing H4a, the indirect effect of perceived usefulness through internet browsing and attitude toward online purchasing was also found to be significant, *b* = 0.02, SE = 0.01, *p* < 0.05, 95% CI = 0.01–0.05, having no zero between confidence interval when both mediating variables were simultaneously entered into the equation.

**Figure 3:**
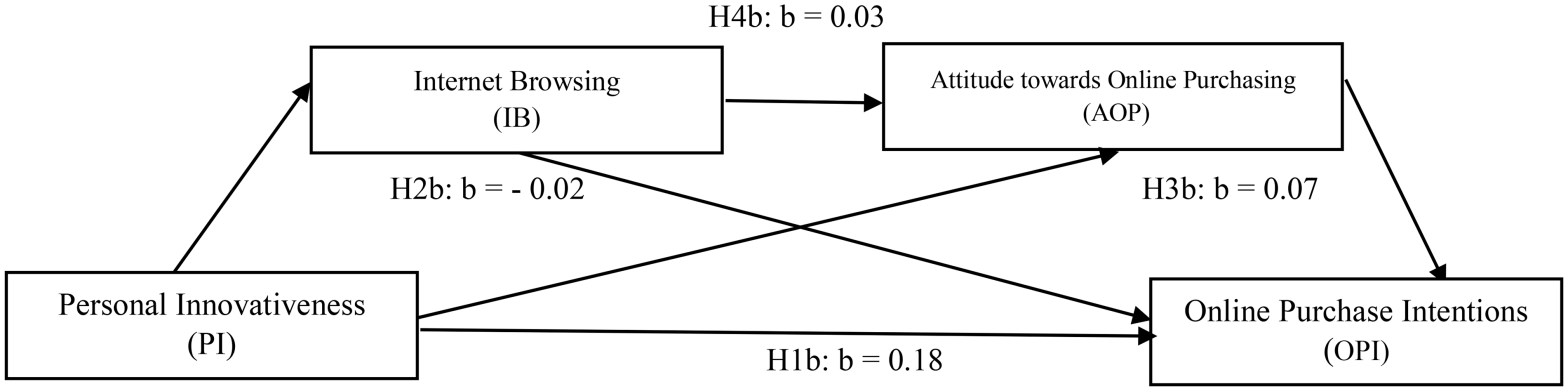
Serial multiple mediation models include Personal Innovativeness (PI), Internet browsing (IB), Attitude toward Online Purchasing (AOP), and Online Purchase Intentions (OPI). Authors constructed.

To test the hypothesis of the proposed model (Figure 2) whether internet browsing and attitude toward online purchasing serially mediated effects of personal innovativeness on online purchase intentions (serial mediation Model 6) by Hayes and Preacher (2013), we used bootstrap methods. Figure 3 illustrates all possible paths and their related coefficient values. We found that the first path (H1b) significantly affected online purchase intentions with coefficient value, *b* = 0.18, *p* < 0.001 and supported H1b.

The statistical significance of the indirect effects in the model tested in the current research was examined on 5,000 bootstrap samples. Estimates were taken within a 95% confidence interval, and bias-corrected results are presented in Table 3. It shows that internet browsing mediated significantly between personal innovativeness and online purchase intentions, *b* = −0.02, SE = 0.02, 95% CI = −0.06, and −0.01 (H2b supported). Likewise, the indirect effect through Attitude toward online purchasing between personal innovativeness and online purchase intentions was significant, *b* = 0.07, SE = 0.02, 95% CI = 0.03, and 0.12, which supported H3b. For testing H4b, the indirect effect of personal innovativeness through internet browsing and Attitude toward online purchasing was also found to be significant, *b* = 0.03, SE = 0.01, *p* < 0.05, 95% CI = 0.01–0.05, having no zero between confidence interval when both mediating variables were simultaneously entered into the equation.

## Discussion

The COVID-19 pandemic is an unusual and constantly changing global situation, presenting crucial consequences for the economies (Koch et al., 2020). Since COVID-19 emerged, consumers have mostly shifted toward online purchasing due to the easy accessibility during the pandemic (Pham et al., 2020). For example, Pakistan’s e-commerce rate is reported to be over 35% in the first quarter of the fiscal year 2021 (Dawn, 2021), where internet users showed a growth rate of 21% from 2020 to 2021 (Digital 2021: Pakistan) . Drawing on TRA and TAM, we develop a research framework to understand consumers’ online purchase intention during the COVID-19 pandemic. The proposed framework was tested using 410 online shoppers’ data.

The study results revealed that personal usefulness increases online purchase intentions, which confirms hypothesis H1a of the study. Our finding is consistent with the extant studies, showing that usefulness enhances online purchase intention of retail shopping (Athapaththu and Kulathunga, 2018; Moslehpour et al., 2018). This finding indicates that usefulness is a necessary antecedent for the online purchase intention and when/if a website is useful to buy online products/services they sell, it can greatly enhance shoppers’ purchase intention. Our results further depict that personal innovativeness positively influences online purchase intentions, confirming hypothesis H1b. These findings get support from the prior literature on the online shopping environment (Dewi et al., 2020).

Further, H2a and H2b examined the mediating role of internet browsing on the relationship between perceived usefulness and online purchase intentions (PU → IB → OPI) as well as personal innovativeness and online purchase intentions (PI → IB → OPI), respectively. Our results support H2a and H2b; thus, the study findings significantly contribute to the literature on online shopping by showing how internet browsing plays a crucial role in the relationships between perceived usefulness, personal innovativeness, and online purchase intentions.

The results also suggest that perceived usefulness and personal innovativeness significantly influence consumers’ internet browsing, thereby enhancing their online purchase intentions. Such findings get support from the earlier literature (Khare et al., 2010). Furthermore, the current research showed that perceived usefulness and personal innovativeness impact online purchase intentions through the consumer’s attitude toward online shopping, confirming hypotheses H3a and H3b. Perceived usefulness and personal innovativeness impact consumers’ attitudes toward online purchases through increased acceptance of technology (O’cass and Fenech, 2003; y Monsuwé et al., 2004), which increases their intentions toward online purchasing (Chiu et al., 2005).

The results further confirm the hypotheses, H4a and H4b, which suggest that perceived usefulness and personal innovativeness enhance consumers’ browsing behavior that develops their toward online shopping, and this attitude toward online shopping ultimately increases their online purchase intentions.

Additionally, the study reveals that the direct impact of perceived usefulness and personal innovativeness is stronger than the indirect effect where a consumer goes through the stages of internet browsing and attitude toward online purchase before moving to the final step of online purchase intentions. This may be due to the reason that in a society like Pakistan, people have more trust in websites (Kouser et al., 2018), and thus if they find perceived usefulness and personal innovativeness toward purchasing, their online purchase intentions would be high due to high level of trust. It may also be because people in Pakistan are inclined to purchase from known shops (Chaudary et al., 2014), which limits their browsing for other online stores. Hence, when the customers find perceived usefulness and personal innovativeness, they bypass the stages of internet browsing and attitude toward online purchasing and directly move to the online purchase intentions.

Furthermore, earlier studies reported that attitude toward online purchases shapes browsing behavior (Seock and Norton, 2007), which is contradictory to the current study’s findings where internet browsing forms the attitude toward online purchases. These findings are similar to the findings of Childers et al. (2001), where web navigation leads to the attitude toward online shopping through ease of use (Childers et al., 2001). However, to the best of our knowledge, no earlier research has studied the serial mediation effect of internet browsing and attitude toward online purchase on online purchase intentions.

## Conclusion

The shifts established during the COVID-19 pandemic appear to stay for a longer period (Koch et al., 2020), which posits serious implications for brick-and-mortar stores due to the rapid growth in digitization. These shifts have changed the consumer’s behavior as well. Hence, the current study highlighted the factors which significantly impact consumer behavior, specifically in the times of this Pandemic (Ait Youssef et al., 2020). The current study examined the direct and indirect effects of perceived usefulness and personal innovativeness on online purchase intentions. The study provides important insights into the role of browsing and attitude in enhancing online purchase intentions, which can serve as an important tool for retailers in improving their online business.

This study reveals significant theoretical implications. Firstly, this study proposed an analytical framework integrating TAM and TRA to study antecedents of online purchase intentions during COVID-19. Additionally, the study extended the implication of theory (e.g., TAM) in the online shopping environment, particularly in a developing country, since most of the earlier studies on TAM have been conducted in developed countries (e.g., Alsabawy et al., 2016; Ashfaq et al., 2020) and no study so far used TAM as a theoretical model to investigate the factor affecting online shopping intentions during this special time. Secondly, the study contributes to the literature by explicitly analyzing the role of internet browsing, which is not thoroughly investigated in the online retail environment (Mallapragada et al., 2016). Thirdly, the study explains the serial mediation mechanism whereby perceived usefulness and personal innovativeness enhance online purchase intentions through internet browsing and attitude toward online purchases, where most of the existing studies showed the direct impact of perceived usefulness and attitude on intentions (Ashfaq et al., 2020, 2021).

The purchasing behavior of online buyers exhibits a significant role in the success of online retailers (Kiang and Shang, 2015). The adoption of online purchasing is one of the major aspects of e-commerce, and developing countries like Pakistan are trying to move toward a digital economy. The social distancing and lockdown approaches have created a surge in this shift toward online purchasing. The current study suggests that online vendors effectively use the findings of the study to enhance consumers’ online purchase intentions. Online retailers can use the outcomes of the current study to build well-structured online shopping platforms along with useful e-marketing strategies to create innovation and usefulness for consumers. Online retailers can increase the perceived usefulness of consumers by improving the online services in terms of easy ordering, convenience in delivery, and providing online consulting services (Moslehpour et al., 2018), which can shape the browsing behavior and ultimately the attitude toward purchase. In addition to utilitarian incentives, firms also need to incent consumers with hedonic values like personal innovativeness. Online retailers need to design their store layout with a novel, innovative and comfortable technology through which consumers high on personal innovativeness will be more likely to display an online purchase behavior (Dewi et al., 2020).

Furthermore, internet browsing is considered an important element for creating a positive attitude toward online shopping. Hence, the current study provides retailers and marketers insight into developing e-business strategies for increasing consumers’ online purchase intentions in conjunction with internet browsing. Online retailers thus need to come up with smart functionalities of their online presence that can help the buyers to have easy accessibility.

Like other research, this study is also not without limitations. Firstly, we used convenience sampling. The data were collected through an online survey due to the COVID-19 pandemic. Secondly, the data was collected from the residents of Pakistan. Hence, it might not represent the general population of other developing countries. Thirdly, the study is constructed employing a cross-sectional approach, and therefore, it is time-dependent. Future researchers may use more diversified samples to increase the validity and generalizability of current study findings. Future research may extend this model by shedding light on the additional factors which can impact browsing and thus increase online purchase intentions. Furthermore, future researchers may focus on comparing the direct and indirect impact of perceived usefulness and personal innovativeness on online purchase intentions in other developing countries.

## Data availability statement

The original contributions presented in the study are included in the article/supplementary material, further inquiries can be directed to the corresponding author.

## Ethics statement

Ethical review and approval was not required for the study on human participants in accordance with the local legislation and institutional requirements. Written informed consent from the patients/participants OR patients/participants legal guardian/next of kin was not required to participate in this study in accordance with the national legislation and the institutional requirements.

## Author contributions

SN: conceptualization and introduction. HB: methodology and interpreted results. MB: literature review and discussion. MA: review and editing. All authors listed have made a substantial, direct and intellectual contribution to the work, and approved it for publication.

## Conflict of interest

The authors declare that the research was conducted in the absence of any commercial or financial relationships that could be construed as a potential conflict of interest.

## Publisher’s note

All claims expressed in this article are solely those of the authors and do not necessarily represent those of their affiliated organizations, or those of the publisher, the editors and the reviewers. Any product that may be evaluated in this article, or claim that may be made by its manufacturer, is not guaranteed or endorsed by the publisher.
